# Two storage hexamerins from the beet armyworm *Spodoptera exigua*: Cloning, characterization and the effect of gene silencing on survival

**DOI:** 10.1186/1471-2199-11-65

**Published:** 2010-08-31

**Authors:** Bin Tang, Shigui Wang, Fan Zhang

**Affiliations:** 1 Hangzhou Key Laboratory of Animal Adaptation and Evolution, College of Life and Environmental Sciences, Hangzhou Normal University, Hangzhou, Zhejiang 310036, China; 2 Institute of Plant and Environment Protection, Beijing Academy of Agriculture and Forestry Sciences, Beijing 100089, China

## Abstract

**Background:**

In insects, hemocyanin superfamily proteins accumulate apparently to serve as sources of amino acids during metamorphosis, reproduction and development. Storage hexamerins are important members of the hemocyanin superfamily. Although insects possess storage hexamerins, very little is known about the character and specific functions of hexamerin 1 and storage protein 1 in insect development.

**Results:**

To gain insight into the function of storage proteins in insects, cDNAs for two storage proteins were cloned from the fat body of *Spodoptera exigua*. *S. exigua *hexamerin 1 (*SeHex*) cDNA contained an open reading frame of 2124 nucleotides encoding a protein of 707 amino acids with a predicted molecular weight of 82.12 kDa. *S. exigua *storage protein 1 (*SeSP1*) cDNA contained an open reading frame of 2256 bp encoding a protein of 751 amino acids with a predicted molecular weight of ~88.84 kDa. Northern blotting analyses revealed that *SeHex *mRNA is expressed in the fat body, cuticle, midgut and Malpighian tubules and *SeSP1 *in fat body, Malpighian tubules and tracheae. *SeHex *and *SeSP1 *mRNAs were expressed in fat body at different levels from first instar larvae to pupae, with expression was much lower from first instar larvae to first-day fifth instar larvae. *SeHex *transcript expression was high in fat body of wandering larvae (pre-pupae) and steadily decreased to the seventh pupal day. *SeSP1 *transcript expression was high in fat body of wandering larvae, 2-day-old fifth instar larvae and 2-, 4- and 7-day-old pupae. *SeHex *and *SeSP1 *mRNAs levels were expressed lower than control on the condition of starvation at 12 h. Of insects injected with SeHex and SeSP1 dsRNA, 38.7% and 24.3% survived to 204 h after treatment, respectively. This was significantly lower than in the controls groups.

**Conclusions:**

These findings provide new data on the tissue distribution, expression patterns and the function in starvation of storage proteins. RNA interference results revealed that storage protein genes are key in metamorphosis, reproduction and insect development. The results for *SeHex *and *SeSP1 *interference reveal that a potential method to control this pest is to disrupt the regulation of storage proteins.

## Background

In insects, hemocyanin superfamily proteins accumulate apparently to serve as sources of amino acids during metamorphosis, reproduction and periods when food is unavailable and the demand for amino acids is high [1,2]. Before the initiation of insect metamorphosis and reproduction, these proteins are progressively stored in larval hemolymph. Hexamerins are mainly synthesized by the fat body during larval development, stored in the hemolymph, and sequestered in the fat body, where they serve as sources of nitrogen and amino acids for pupae and adults during metamorphosis and reproduction [3-5].

The insect tracheal system has many respiratory proteins that transport oxygen in the hemolymph and, in preparing for metamorphosis, insect larvae store a huge amount of protein in hemolymph [6-9]. These specialized oxygen-transport proteins evolved from the copper-containing hemocyanins [10]. In most Hexapoda, gas exchange is mediated by the tracheal system, a network of tubules that open to the atmosphere on the cuticle and radiate to all parts of the body. Oxygen is delivered through the trachea and tracheoles in the gaseous phase [11]..

Insect hexamerins share sequence similarities with other proteins of divergent functions [12] and belong to a protein superfamily that also comprises arthropod phenoloxidases, crustacean pseudohemocyanins, insect storage hexamerins and dipteran hexamerin receptors [10,12-15]. The most abundant and widely distributed storage proteins that accumulate in the hemolymph or fat body are composed of six identical or similar subunits of ~80 kDa, and thus are also called hexamerins [4,13,16-18]. Storage hexamerins include the hexamerins, juvenile hormone-related protein, riboflavin-binding hexamerin precursor, methionine-rich storage protein (storage protein 1, SP1), very-high-density lipoprotein, tyrosine-rich proteins and hemocyanin-related proteins [4,19].

Genes for storage hexamerins have been cloned from plants, bacteria and fungi. Gordadze *et al. *cloned two hexamerin cDNAs from the mosquito *Aedes aegypti *[20]. So far, SPs and hexamerins have been cloned and studied in *Musca domestica *[21], *Apriona germari *[22,23], *Apis mellifera *[5], *Drosophila melanogaster *[24] and *Reticulitermes flavipes *[25]. In Lepidoptera, SPs and hexamerins have been cloned and studied in *Spodoptera litura *[26], *Plodia interpunctella *[2], *Corcyra cephalonica *[27], *Manduca sexta *[28], *Amsacta albistriga *[29-31], *Plutella xylostella *[32], *Helicoverpa zea *[33], *Omphisa fuscidentalis *[34], and *Sesamia nonagrioides *[35,36].

*Spodoptera exigua*, commonly called the beet armyworm, is a worldwide agricultural pest that has developed resistance to many chemical pesticides. In the present study, we cloned cDNA for two hexamerin genes from *S. exigua *(EF646282 and EU259816). We observed expression of these two genes not only in fat body, but also in Malpighian tubules and other tissues. Furthermore, the genes exhibited differential expression patterns in fat body and they play a role in starvation. We used RNA interference (RNAi) to investigate the functions of the genes.

## Results

### *SeHex *and *SeSP1 *cDNA sequence analysis

**Figure 1:**
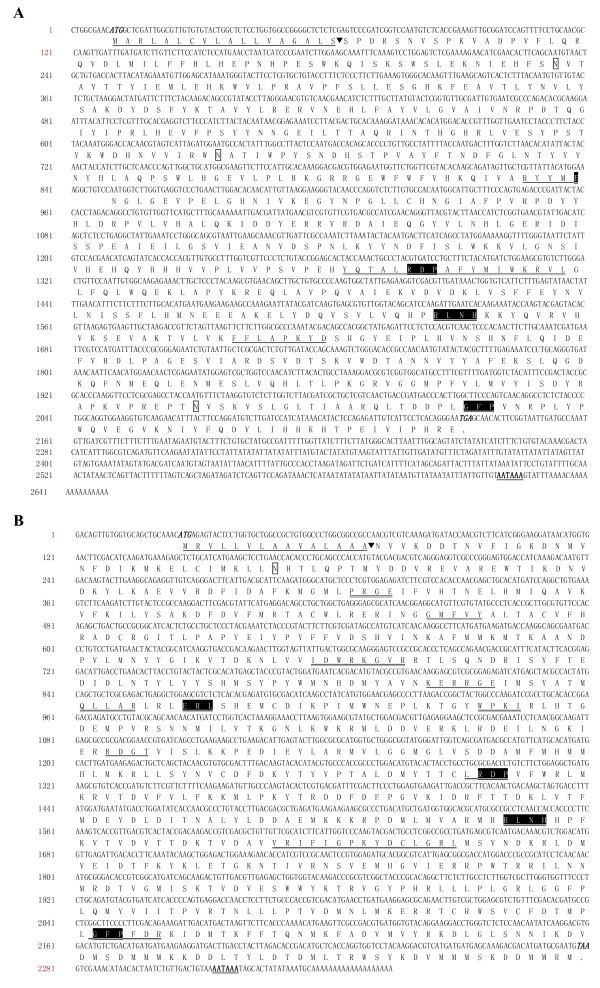
**Nucleotide and deduced amino acid sequences for *S. exigua Hex *and *SP1 *cDNAs**. Italic and bold nucleotides indicate the start and stop codons, respectively. The termination signal AATAAA is bold and underlined. The hemocyanin group of motifs (or signature motifs) (amino acid residues ERL, RDP, RLNH and GFP) are shaded in black. **(A) ***SeHex *cDNA sequence analysis. Underlined amino acid residues (1-18) and the arrowhead represent the signal peptide and putative cleavage site, respectively. Regions that are highly conserved in lepidopteran *Hex *genes are double underlined. Potential N-glycosylation sites (residues 75, 209, 479 and 647) are boxed. The nucleotide sequence reported in this paper has been submitted to GenBank (accession number EF646282). **(B) ***SeSP1 *cDNA sequence analysis. Underlined amino acid residues (1-15) and the arrowhead represent the signal peptide and putative cleavage site, respectively. Regions that are highly conserved in lepidopteran *SP1 *genes are double underlined. The potential N-glycosylation site (residue 47) is boxed. The nucleotide sequence reported in this paper has been submitted to GenBank (accession number EU259816).

*SeHex *had an open reading frame of 2124 bp, encoding a protein of 707 amino acids (Figure 1A) with a predicted molecular weight (MW) of approximately 82.12 kDa and an isoelectric point (p*I*) of 6.48. *SP1 *had an open reading frame of 2256 bp, encoding a protein of 751 amino acids (Figure 1B) with a predicted MW of approximately 88.84 kDa and p*I *of 9.10.

**Figure 2:**
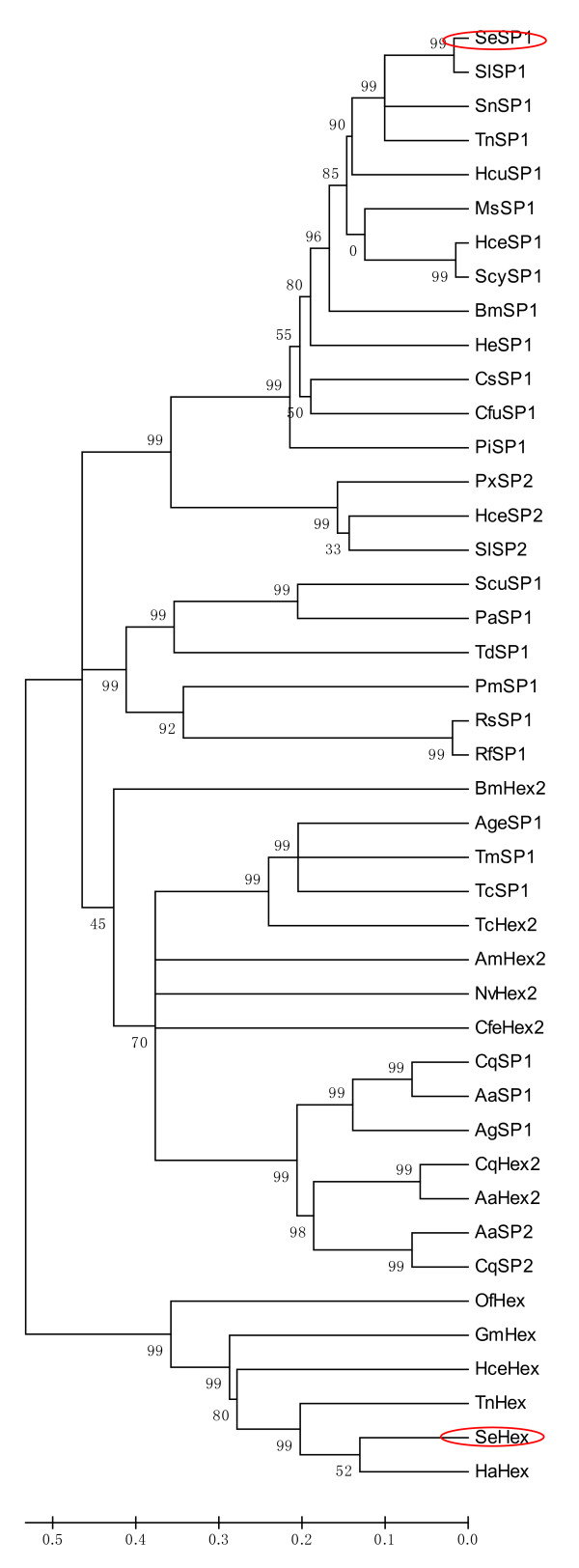
**Phylogenetic analysis of insect hemocyanins based on their amino acid sequences**. Full-length amino acid sequences were aligned using Mega 3.1 to generate a phylogenetic tree (Hex, hexamerin; Hex2, class 2 hexamerin gene; SP1, storage protein 1; SP2, storage protein 2). A bootstrap analysis was carried out and the robustness of each cluster was verified in 1000 replications. The hemocyanins were from *Aedes aegypti *(*AaHex2*, GALLHP82 and *AaSP1*, XM_001659481 and *AaSP2*, XM_001659481), *Anopheles gambiae *(*AgSP1*, XM_321800), *Apis mellifera *(*AmHex2*, NM_001011600), *Apriona germari *(*AgeSP1*, AF509880), *Bombyx mori *(*BmHex2*, NM_001044125 and *BmSP1*, NM_001113276), *Camponotus festinates *(*CfeHex2*, AJ251271), *Chilo suppressalis *(*CsSP1*, AB248057), *Choristoneura fumiferana *(*CfuSP1*, AF007768), *Culex quinquefasciatus *(*CqSP1*, XM_001843442 and *CqSP2*, XM_001843444), *Galleria mellonella *(*GmHex*, GALLHP82X;), *Heliconius erato *(*HeSP1*, EU711403), *Helicoverpa armigera *(*HaHex*, AY661710), *Hyalophora cecropia *(*HceHex*, AF032397 and *HceSP1*, AF032399 and *HceSP2*, AF032398), *Hyphantria cunea *(*HcuSP1*, U60988), *Manduca sexta *(*MsSP1*, L07609), *Nasonia vitripennis *(*NvHex2*, XM_001606979), *Omphisa fuscidentalis *(*OfHex*, EF429085), *Periplaneta americana *(*PaSP1*, FM242648), *Perla marginata *(*PmSP1*, AM690365), *Plodia interpunctella *(*PiSP1*, AF356843), *Plutella xylostella *(*PxSP2*, AB266596), *Reticulitermes flavipes *(*RfSP1*, AY572858), *Reticulitermes speratus *(*RsSP1*, AB371986), *Samia cynthia *(*ScySP1*, AB288051), *Sesamia nonagrioides *(*SnSP1*, DQ147770), *Sinella curviseta *(*ScuSP1*, FM242638), *Spodoptera exigua *(*SeHex*, EF646282 and *SeSP1*, EU259816), *Spodoptera litura *(*SlSP1*, AJ249470; *SlSP2*, AJ249468), *Tenebrio molitor *(*TmSP1*, AF395329), *Thermobia domestica *(*TdSP1*, FM165290), *Tribolium castaneum *(*TcHex2*, XM_968706 and *TcSP1*, XM_967951) and *Trichoplusia ni *(*TnHex*, CBLJHSP and *TnSP1*, L03280).

*SeHex *cDNA (GenBank accession no. EF646282) has 43-74% identity to other known *Hex *genes (Additional file 1). *SeHex *is most similar to *Hex *from the lepidopteran *H. armigera *(74% identity). It is also similar to *Hex *genes from *Trichoplusia ni*, *Hyalophora cecropia*, *Galleria mellonella *and *O. fuscidentalis *(Figure 2). *SeSP1 *cDNA (GenBank no. EU259816) has 28-96% identity to other known *SP1 *genes (Additional file 1). *SeSP1 *is most similar to *SP1 *from the lepidopteran *S. litura *(96% identity). It is also similar to *SP1 *genes from *T. ni*, *S. nonagrioides*, *H. cecropia*, *Hyphantria cunea*, *M. sexta*, *Samia cynthia*, *Heliconius erato*, *Bombyx mori*, *Chilo suppressalis*, *P. interpunctella*, *A. aegypti*, *Culex quinquefasciatus*, *Anopheles gambiae*, *Periplaneta americana*, *Perla marginata*, *Thermobia domestica*, *Reticulitermes speratus*, *A. germari*, *Sinella curviseta*, *Tribolium castaneum*, *Tenebrio molitor *and *R. flavipes *(Figure 2).

### Protein sequence analysis

In Figure 1, underlined amino acid residues 1-18 and 1-15 represent the signal peptides for *SeHex *and *SeSP1*, respectively. The deduced amino acid sequences for *SeHex *and *SeSP1 *contain not only four signature motifs of the hemocyanin family, such as ERL (residues 277-279 and 281-283), RDP (424-426), RLNH (506-509) and GFP (668-670 and 630-632), but also other conserved motifs. Sequence alignment for lepidopteran Hex proteins revealed three highly conserved regions (Additional file 1A). Alignment of lepidopteran SP1 proteins revealed ten highly conserved motifs (Additional file 1B). Four potential N-glycosylation sites (amino acids 75, 209, 479 and 647) were found in *SeHex*, but only one potential site (amino acid 47) in *SeSP1 *(Figure 1, Additional file 1).

Results for two hexamerin genes from the termite *R. flavipes *(*RfHex*) revealed conserved hexamerin signature motifs (ADKP/QFKM/QK/RQ, YFTEDVGL and TALRDPAY/NQ) [24]. The protein sequence encoded by *SeHex *had only two signature motifs (YFTNDFGL and TALRDPAFY) in common with RfHex. Sequence alignment revealed four conserved signature motifs (ERL, RDP, RLNH and GFP) for insect hexamerins and storage proteins. Three conserved motifs (RYYMRRLS, YQTALR/SDPAY/FYMIM/WKRVL and FFLAPKYD) were observed for lepidopteran *Hex *genes (Figure 1 and Additional file 1). Moreover, lepidopteran *SP1 *genes had ten highly conserved regions (PRGE, GMFV/LY, I/VDW/SRKGV/LR/P, KERR/QGE, QLLAR, WPKI, RDGT, LRDP, VRI/VFL/IGPKY/FDCM/LGR/LL and GFPFDR) (Figure 1 and Additional file 1).

### Tissue distribution of *Hex *and *SP1 *in *S. exigua*

**Figure 3:**
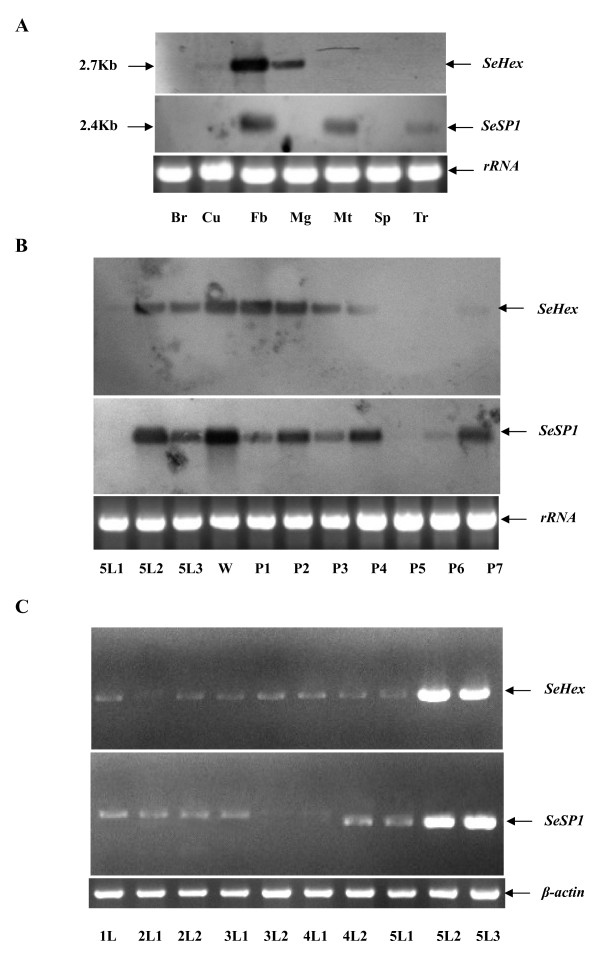
**Northern blotting analyses of *SeHex *and *SeSP1 *transcript expression in fat body**. **(A) **For Northern blotting analysis, total RNA of day-2 fifth instar *S. exigua *larvae was extracted from various tissues: brain (Br), cuticle (Cu), fat body (Fb), midgut (Mg), Malpighian tubules (Mt), spermary (Sp) and tracheae (Tr). **(B) **Developmental expression of *SeHex *and *SeSP1 *was analyzed by Northern blotting of fat body from fifth instar larvae to pupae of *S. exigua*. RNA was extracted from fat body of fifth instar larvae (5L), wandering (pre-pupae) larvae (W) and pupae (P). 5L1-5L3 denoted day-1 to day-3 fifth instar larvae and P1-P7 denote day-1 to day-7 pupae. rRNA was used as a control in all Northern blotting analysis. **(C) **Developmental expression of *SeHex *and *SeSP1 *in young larvae was analyzed by RT-PCR in fat body of *S. exigua*. RNA was extracted from the fat body of first (1L), second (2L), third (3L), fourth (4L) and fifth instar larvae (5L). L1 means the first day of young larvae, and so on. β-Actin was used as a control in all RT-PCTR analyses.

Northern blotting revealed strong *SeHex *bands for fat body and midgut and weak bands for cuticle and Malpighian tubules, but no transcripts were detected for brain, spermary and trachea samples from *S. exigua *larvae (Figure 3A). The results suggest that *SeHex *is specifically expressed in fat body, midgut, cuticle and Malpighian tubules. Northern blotting results for *SeSP1 *transcripts revealed expression in fat body, Malpighian tubules and tracheae, but not in brain, cuticle, midgut and spermary (Figure 3A).

### Developmental expression of *SeHex *and *SeSP1*

*SeHex *and *SeSP1 *mRNAs were expressed in fat body at different levels from fifth instar larvae to pupae. *SeHex *transcripts were highly expressed in fat body in wandering larvae (pre-pupae), as well as in day-1 and day-2 pupae. Transcripts were present at lower levels in fat body of day-2 and day-3 fifth instar larvae and the day-3 and day-4 pupae. *SeHex *transcripts were very low in fat body of day-1 and day-7 pupae (Figure 3B). Moreover, *SeHex *expression steadily decreased from wandering larvae to 6-day-old pupae. The results suggest that *SeHex *mRNA is constitutively expressed at a rather high level in fat body from the second day of larva stages to the fourth day of pupa stages. *SeSP1 *transcripts were highly expressed in fat body in wandering larvae (pre-pupae), as well as in day-2 fifth instar larvae and day-2, day-4 and day-7 pupae. It was present at lower levels on day-3 fifth instar larvae and day-1, day-3 and day-6 pupae (Figure 3B).

Comparative RT-PCR results showed that *SeHex *and *SeSP1 *mRNAs were expressed at very low levels in fat body from first instar larvae to day-1 fifth instar larvae. *SeHex *and *SeSP1 *mRNAs expression was higher in day-2 fifth instar larvae (Figure 3C).

### *SeHex *and *SeSP1 *proteins' function identified on the condition of starvation

**Figure 4:**
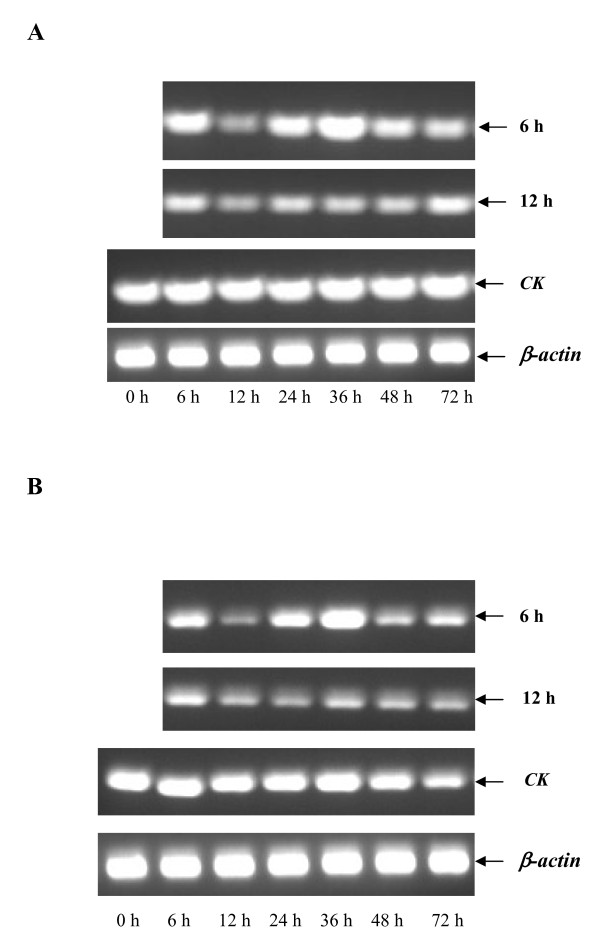
***SeHex *and *SeSP1 *mRNA expression on the condition of starvation**. The day-2 fifth instar larvae were used as experimental insects in the treatment of starvation. In the experiment of starvation, two experimental group insects were hungered for 6 h and 12 h, respectively. Followed these insect continue to rear at 25 ± 1°C with an L14:D10 photoperiod using an artificial diet. The insects were observed at 0 h (before treatment), 6 h, 12 h, 24 h, 36 h, 48 h and 72 h after treatment. Every three to five lively larvae were randomly selected and stored at -80°C for subsequent RNA extraction. **(A) **for SeHex in starvation, **(B) **for SeSP1 in starvation. The housekeeping gene β-actin was used as a reference.

RT-PCR analysis showed that *SeHex *and *SeSP1 *transcripts were mainly lowly expressed when the insects were been on the condition of starvation. And *SeHex *and *SeSP1 *transcripts expressed in the same mode or tread in the different starvation treatment (Figure 4A, 4B). In the group of starvation with 6 h, once stress after finals, *SeHex *and *SeSP1 *mRNA expressional levels lower than control, but higher than the group of starvation with 12 h at 6 h, 24 h and 36 h (Figure 4A, 4B). In the group of starvation with 12 h, once stress was finished, *SeHex *and *SeSP1 *mRNA expressed decreased and keep the lower expressional levels.

### Survival rate and *SeHex *and *SeSP1 *transcript analysis after double-stranded RNA injection

**Figure 5:**
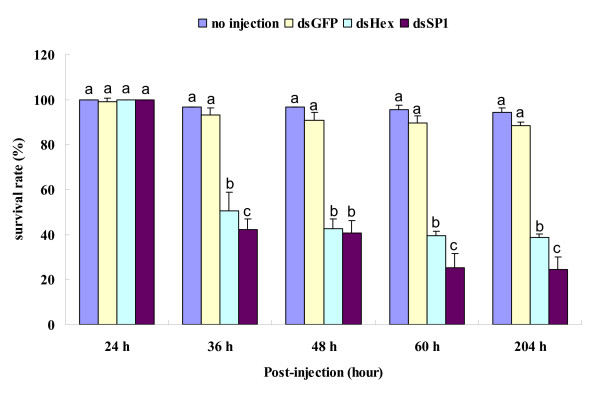
**Survival after injection of *SeHex *and *SeSP1 *dsRNA**. Insect survival rates at different times after injection of dsSeHex, dsSeSP1 and dsGFP. The survival rate was assessed at key developmental stages of 24 h (fifth instar, 3-day-old larvae), 36 h, 48 h (pre-pupae), 60 h (pupae) and 204 h (adult) after injection. Results were arcsine square-root transformed before analysis to correct for the non-normal distribution of percentage values. Different letters at the same detection time indicate a significant difference in survival rate (p < 0.05, Duncan's test). No significant difference was found by ANOVA (p > 0.05). All error bars represent standard deviation (n = 3).

Double-stranded RN**A **for SeHex and SeSP1 (dsSeHex and dsSeSP1) was injected into day-2 fifth instar larvae. The survival rate of insects injected with dsSeHex was 50.55%, 42.60%, 39.42% and 38.73% at 36, 48, 60 and 204 h after injection, respectively, which is significantly lower than the survival of insects in the two control groups (Figure 5). A sharp decrease in survival rate was observed between 24 and 36 h after dsSeHex injection. The survival rate of insects injected with dsSeSP1 was 42.18%, 40.54%, 25.07% and 24.29% at 36, 48, 60 and 204 h after injection, respectively, which is significantly lower than the survival of insects in the two control groups (Figure 5). Two sharp decreases in survival rate were observed from 24 to 36 h and from 48 to 60 h after dsSeSP1 injection. Prior to death, these larvae usually became less vital and smaller in size.

**Figure 6:**
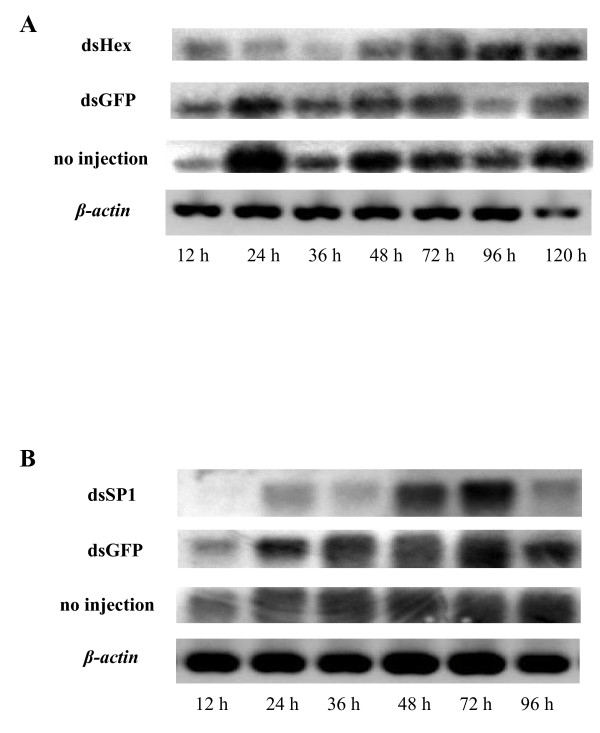
**(A) *SeHex *and (B) *SeSP1 *transcript analysis by RT-PCR amplification after dsRNA injection**. Three larval states (before death, still living and less vital) were randomly selected at each time point after injection. Total RNA was extracted and reversed to cDNA using AMV reverse transcriptase (Takara). *SeHex *and *SeSP1 *specific probes were radiolabeled with [α-^32^P]-dCTP. The specific primers SeHex-FP and SeHex-RP or SeSP1-FP and SeSP1-RP were used to amplify cDNAs in the same PCR reactions. The PCR products were separated on 2% agarose gel and transferred to a Hybond-N^+ ^nylon membrane. Hybridization, washing and signal detection of the blots were carried out as described previously. **(A) **Lanes marked 12 h (5L2), 24 h (5L3), 36 h (5L3), 48 h (W), 72 h (P1), 96 h (P2) and120 h (P3) indicate the times (and developmental stage) after injection. **(B) **Lanes marked 12 h (5L2), 24 h (5L3), 36 h (5L3), 48 h (W), 72 h (P1) and 96 h (P2) indicate the times (and developmental stage) after injection. The housekeeping gene β-actin was used as a reference.

To test the RNAi efficiency, semi-quantitative RT-PCR was performed to detect *SeHex *and *SeSP1 *transcripts. *SeHex *and *SeSP1 *mRNA levels substantially decreased at 12, 24 and 36 h after injection compared to the negative controls (dsGFP and no injection) (Figure 6A, B). However, transcription of both genes recovered to some extent 48 h after injection.

## Discussion

Many different storage proteins are found in larvae and pupae at different stages, but not all species have all the storage proteins. Ryan *et al. *were the first to identify a 74-kDa hexamerin subunit in *A. mellifera *larval hemolymph [37]. Two storage proteins have also been investigated in the honeybee *A. mellifera *[38], the ant *Camponotus festinatus *[39,40], and several other ant species [41]. In Lepidoptera, two different types of methionine-rich hexamerins can be classified according to their common amino acid composition [16,42,43], and three or four SPs are found in some species. Storage proteins first reported in *T. ni *in final-stage larvae [44] and two SPs in *M. sexta *have been separated and cloned [45,46]. Six storage proteins of four different types have been found in *Caplodes ethlius *[19]. In the present study, two storage proteins were cloned from *S. exigua *(Figure 1). The results in Figure 2 show that three storage proteins (SP1, SP2 and Hex2) are found in the Hymenoptera *A. aegypti *and *C. quinquefasciatus*. Moreover, no more than three different storage proteins have been found in Lepidoptera (Figure 2).

Storage proteins serve as a source of amino acids for tissue metamorphosis during pupal development and have been shown to be a component of the sclerotizing system of the cuticle [47]. They also serve as an ecdysteroid carrier in the hemolymph and function in nutrient uptake and storage; some are also capable of binding the insect morphogenetic hormone juvenile hormone [48-50]. Storage proteins are mainly synthesized in fat body during larval development and stored in the hemolymph [9]. *Corcyra cephalonica *hexamerin protein 2 was expressed in fat body and carcass tissue, but not in salivary gland, midgut or Malpighian tubules [27]. Kim *et al. *reported that *A. germari *hexamerin was expressed not only in fat body, but also in midgut, in agreement with our results for *SeHex1 *[22]. We found that *SeHex *was expressed not only in fat body and midgut, but also in cuticle and Malpighian tubules (Figure 3A). SP1 in *Amsacta albistriga *was detected in fat body and pupal ovary [31]. However, the results for SeSP1 reveal that it is expressed in fat body, Malpighian tubules and tracheae (Figure 3A). Storage proteins do not bind copper or transport oxygen to the hemolymph [51,52], and lower expression levels were detected in Malpighian tubules and tracheae (Figure 3A). This is consistent with the theory of hemocyanin superfamily evolution.

Storage proteins accumulate in large quantities in hemolymph during final instar larvae, are taken up by fat body cells and serve as a reservoir for subsequent development [4,43,53]. *CcHex2 *mRNA was present at all stages of larval development of C. *cephalonica *and reached a maximum in fat body of final instar larvae [27]. *AalSP1 *expression gradually increased from day-1 to day-7 final instar larvae [30]. It has been reported that expression levels of insect storage proteins (including Hex1, Hex2, SP1 and SP2) reach a maximum in the last stage of final instar larvae [31,54]. *SeHex *and *SeSP1 *mRNA expression also reached a maximum in the final stage (pre-pupae) of last instar larvae (Figure 3B). However, *SeHex *and *SeSP1 *mRNA expression was much lower before the first day of fifth instar larvae, so it was not detected in the 25 μg of total RNA used for Northern blotting. In order to get the exact expression patterns of SeHex and SeSP1 mRNA in the larvae developmental stages, the more sensitive way of RT-PCR is used. And RT-PCR results confirmed that expression of these storage proteins is high in final instar larvae (Figure 3C). This result showed that storage protein can be accumulated on the good nutritional status, just as the last larvae in *S. exigua*.

The function of storage proteins is clear. In *M. sexta*, starvation is effective in reducing mRNA levels of both hexamerin genes [55]. In the group of starvation with 12 h, *SeHex *and *SeSP1 *mRNA expression was much lower than control (Figure 4A, 4B), that showed storage proteins are key in the stress of starvation. Hakim and colleagues reported that a monomeric α-arylphorin storage protein can stimulate stem cell proliferation in the lepidopterans *M. sexta *and *Spodoptera littoralis *and in the beetle *Leptinotarsa decemlineata*. In addition, feeding an artificial diet containing arylphorin increased the growth rate of several insect species [56]. It is known that storage proteins are crucial for insect development and disruption has a negative effect on the natural development and movement of an insect, which may eventually die. RNAi was used to investigate the function of *SeHex *and *SeSP1 *in the present study and resulted in adult survival rates of 38.73% and 24.29%, respectively (Figure 5). Moreover, *SeHex *and *SeSP1 *mRNA was substantially lower at 36 h after injection compared to the negative control (Figure 6). And the expression levels of some control larvae are weird may result in the side effect of inject. The results confirm that *SeHex *and *SeSP1 *mRNA expression is crucial for insect life. Abnormal morphological development was not observed after RNAi treatment. It is also found that *SeHex *and *SeSP1 *mRNA are lower on the condition of starvation. Hence, a lack of nutritional reserves or developmental failure may be key reasons for the lethality. Nevertheless, the *SeHex *and *SeSP1 *RNAi results suggest that a potential method for control of this pests is to disrupt the regulation of storage proteins.

## Conclusions

We demonstrated that genes for two storage hexamerins exist in *S. exigua*. *SeHex *transcripts were expressed not only in fat body, but also in cuticle, midgut and Malpighian tubules. *SeSP1 *mRNA was expressed in fat body, Malpighian tubules and tracheae. Furthermore, *SeHex *and *SeSP1 *have differential expression patterns in fat body. The results of starvation treatment suggest that *SeHex *and *SeSP1 *have important functions in *S. exigua*. RNAi results showed that these proteins are critical for *S. exigua *metamorphosis, indicating that a potential method for control of this pest is to disrupt the regulation of storage proteins.

## Methods

### Insect culture

*S. exigua *larvae were reared at 25 ± 1°C with an L14:D10 photoperiod on an artificial diet [37]. The developmental stages were synchronized at each molt by collecting new larvae or pupae. The brain, midgut, fat body, cuticle, Malpighian tubules, spermary and tracheae from different stages from fifth instar larvae and the fat body from day-1 fifth instar to day-7 pupae were dissected in saline containing 0.75% NaCl and stored at -80°C until further use.

### RNA isolation and cDNA synthesis

Total RNA was isolated from the fat body of *S. exigua *pupae using the acid guanidinium thiocyanate-phenol-chloroform method [57,58]. The fat body (100 mg) was homogenized in solution D (4 M guanidinium thiocyanate; 25 mM sodium citrate, pH 7; 0.5% sarcosyl; 0.1 M 2-mercaptoethanol), placed on ice for 5 min, and then sodium acetate, phenol (pH 7.0) and chloroform/isoamyl alcohol (49:1) were added. The mixture was centrifuged at 10,000×*g *at 4°C for 20 min. The supernatant was transferred to a new tube and then isopropanol was added. After centrifugation, the RNA pellet was washed with 75% ethanol and then dissolved in ddH_2_O. A sample of 1 μg of total RNA was reverse transcribed at 42°C for 1 h in a 25- μl reaction solution containing reaction buffer, 10 mM DTT, 0.5 mM dNTPs, 0.5 μg of oligo-dT18 and reverse transcriptase from avian myeloblastosis virus (AMV, Takara, Japan) [59].

### Polymerase chain reaction and rapid amplification of cDNA ends

Two pairs of degenerate primers were designed based on the conserved amino acid sequences of known Hex and SP1 proteins. The first PCR reaction was performed with primers DF1 and DR1 using the following conditions: three cycles of 30 s at 94°C, 30 s at 45°C and 90 s at 72°C, followed by 30 cycles of 30 s at 94°C, 30 s at 48°C and 90 s at 72°C. A second PCR was carried out using the nested primers DF2 and DR2 using the same cycle conditions [60]. The products were subjected to agarose electrophoresis and the DNA bands were then excised from the gel and purified using a DNA gel extraction kit (Omega, USA). The PCR products were cloned into the pMD18-T vector (Takara) and sequenced by the dideoxynucleotide method (Invitrogen).

cDNA was synthesized according to the manufacturer's protocol (SMART™ kit, Clontech/Takara). Specific primers, Hex-5R1, Hex-5R2, SP1-5R1 and SP1-5R2 for 5'-RACE and Hex-3F1, Hex-3F2, SP1-3F1 and SP1-3F2 for 3'-RACE (Table 1), were designed and synthesized based on the cDNA sequence of the known PCR fragment. 5'-RACE was performed using 2.5 μl of 5'-ready-cDNA with Universal Primer Mix (UPM, Clontech/Takara) and Hex-5R1 or SP1-5R1. Then nested PCR was carried out using Nested Universal Primer A (NUP, Clontech/Takara) and Hex-5R2 or SP1-5R1. 3'-RACE was performed using 2.5 μl of 3'-ready-cDNA with UPM and Hex-3F1 or SP1-3F1, then with NUP and Hex-3F2 or SP1-3F2. The PCR conditions were as follows: 10 min at 94°C, followed by 35 cycles of 30 s at 94°C, 30 s at 60°C and 150 s at 72°C, then 10 min at 72°C. Samples were then kept at 12°C.

**Table 1:** Primers for cDNA cloning, Northern blotting, semi-quantitative RT-PCR, dsRNA synthesis and detection of *SeHex *and *SeSP1 *RNAi

PCR Fragme	Primers			
	
	Name	Direction^a ^	Type^b^	Nucleotide Sequence (5'-3')
1	Hex-DF1	F	D	TGG ARM GHC TGT CYA ACG
	Hex-DF2	F	D	ACA AKG GMA TBH HST TCC
	SP1-DF1	F	D	GGC ATG TTC DTV TAT GC
	SP1-DF2	F	D	TAC CCN TAC TWC TTC GTC
	Hex-DR1	R	D	GAA BGG CAT RCC DCC GAC
	Hex-DR2	R	D	GTC CAG CTS SAW GAA GTT
	SP1-DR1	R	D	CGT ACT TGG GNC CAN DGA AG
	SP1-DR2	R	D	TGA ANG GVT GRT GRT TGA G
2 RACE	3-CDS	R	O	AAG CAG TGG TAT CAA CGC AGA GTA C(T)_30_VN
	NUP	F	A	AAG CAG TGG TAT CAA CGC AGA GT
	Hex-5R1	R	G	GCT GAT GTC AAT ACG TTC ACC GAG
	Hex-5R2	R	G	CGT AAC CCT GTT CGA TGG CGT CAC
	SP1-5R1	R	G	GAT GAC ATG GCT ATC GAC GA
	SP1-5R2	R	G	CGT AGG GAG CAG GCA GAG TG
	Hex-3F1	F	G	GTG AGC GTG TTG GTA CAG CAT CC
	Hex-3F2	F	G	GAG TGA AGT TGC TAA GAC CGT TC
	SP1-3F1	F	G	CAA CTG ACA AGC TAG TGA CC
	SP1-3F2	F	G	CGC TGA GAT GAA GAA GAA GC
3 probe	Hex-FP	F	G	CGT TTG CAC GAG GTC TTC C
	Hex-RP	R	G	GGC TTC TTC TTC ATT CAT GTG C
	SP1-FP	F	G	GAC CAA GGC AGC GAA TGA CC
	SP1-RP	R	G	CAG GGC GTT GGT GAT ATC CA
4 dsRNA	dsHex-F	F	G	GAC AGC CAC GGC TAT GAG ATT CC
	dsHex-FT	F	G	GGA TCC TAA TAC GAC TCA CTA TAG GNG ACA GCC ACG GCT ATG AGA TTC C
	dsHex-R	R	G	CCT TAG AAA CAT TGG TAG GCT CG
	dsHex-RT	R	G	GGA TCC TAA TAC GAC TCA CTA TAG GNC CTT AGA AAC ATT GGT AGG CTC G
	dsSP1-F	F	G	GAC TGG AAA GAA CAC CAT CGT C
	dsSP1-FT	F	G	GGA TCC TAA TAC GAC TCA CTA TAG GNG ACT GGA AAG AAC ACC ATC GTC
	dsSP1-R	R	G	GGT CCT TCC TGT ACA CCA TCA C
	dsSP1-RT	R	G	GGA TCC TAA TAC GAC TCA CTA TAG GNG GTC CTT CCT GTA CAC CAT CAC

^a ^F, forward, R, reverse; ^b ^D, degenerate primer; G, gene-specific primer; A, nested universal primer; O, 3'-RACE CDS primer.

### Cloning of full-length *SeHex *and *SeSP1 *cDNA

Based on the conserved amino acid and nucleotide sequences of hexamerin and storage protein 1 from *B. mori*, *A. aegypti*, *A. gambiae*, *G. mellonella*, *Helicoverpa armigera *and *M. sexta*, we designed eight degenerate primers (Hex-DF1, Hex-DF2, Hex-DR1, Hex-DR2, SP1-DF1, SP1-DF2, SP1-DR1and SP1-DR2) for PCR reactions. Fragments of 800 bp (SeHex-DF2 and SeHex-DR2) and 1100 bp (SeSP1-DF2 and SeSP1-DR2) were first obtained from fat-body cDNA by a second PCR. After sequencing, the deduced amino acid sequence was found to have a high degree of similarity to insect hexamerin classes. We then performed 5' and 3' rapid amplification of cDNA ends (RACE) using several specific primers (Table 1) designed based on the fragment sequences, as well as universal primers (Clontech). The 600- and 1000-bp PCR products for *SP1 *and two 1100-bp PCR products for *Hex *were amplified by 5' and 3'-RACE, respectively. After assembling the overlapping fragments, full-length cDNAs of 2651 bp for *Hex *and 2254 for *SP1 *were obtained.

### Analysis of *SeHex *and *SeSP1 *cDNA sequences

The neighbor-joining method was used to construct a phylogenetic tree with MEGA 3.1 software based on the amino acid sequences of known insect hexamerins. *SeHex *and *SeSP1 *cDNA sequences were compared with those for other Hex, SP1 or hexamerin proteins deposited in GenBank using the BLAST-N or BLAST-X tools at the National Center for Biotechnology Information (NCBI) website. The amino acid sequences encoded by *SeHex *and *SeSP1 *were deduced from the corresponding cDNA sequences using the translation tool at the ExPASy Proteomics website http://expasy.org/tools/dna.html. A bootstrap analysis was carried out and the robustness of each cluster was verified using 1000 replicates. Other protein sequence analysis tools used in this study, including MW, *p*I and N-glycosylation sites, were obtained from the ExPASy Proteomics website http://expasy.org/. Multiple sequence alignment of insect hemocyanin family protein sequences was performed using the tool at the multiple sequence alignment website http://bioinfo.genotoul.fr/multalin/multalin.html.

### Probe labeling and Northern blotting analysis

Tissue-specific expression of *SeHex *and *SeSP1 *was determined by Northern blotting. cDNA fragments of 990 bp (primers Hex-FP and Hex-RP) and 895 bp (primers SP1-FP and SP1-RP) were labeled with [α-^32^P]dCTP using a random primer DNA labeling kit (Takara, Japan) as the hybridization probe [59]. Samples of 25 μg of total RNA isolated from brain, midgut, fat body, cuticle, Malpighian tubules, spermary and tracheae of day-3 fifth instar larvae were separated on a formaldehyde agarose gel containing ethidium bromide [59]. The RNA was subsequently blotted onto a Hybond-N^+ ^membrane (Amersham) and pre-hybridized at 42°C for 3-6 h. The [α-^32^P]-labeled *SeHex *and *SeSP1 *probes were added and incubated at 42°C for up to 12 h in 5× SSPE (0.75 M NaCl, 0.05 M NaH_2_PO_4_, 0.005 M EDTA, pH 7.4) containing 50% formamide, 5× Denhardt's solution, 0.1% SDS and 100 mg/ml salmon sperm DNA [61,62]. After hybridization, the membrane was washed with 0.2× SSPE (0.03 M NaCl, 0.002 M NaH_2_PO_4_, 0.0002 M EDTA, pH 7.4) at 45°C and exposed to X-ray film at -70°C for 24 h [59].

### Developmental expression of *SeHex *and *SeSP1 *by Northern blotting and RT-PCR amplification

Developmental expression of *SeHex *and *SeSP1 *from fifth instar larvae to pupae was determined by Northern blotting. The fat body was dissected from day-1 fifth instar larvae to day-7 pupae. Total RNA was isolated from the fat body and 25 μg was separated on a formaldehyde agarose gel containing ethidium bromide. Northern blotting was carried out as described above. A sample of 25 μg of total RNA was also used to detect the expression of *SeHex *and *SeSP1 *in young larvae, but the results were poor. Thus, more sensitive RT-PCR was used to detect the expression of *SeHex *and *SeSP1 *in larval stages. The PCR reaction was performed with primers Hex-FP and Hex-RP (or SP1-FP and SP1-RP) using the following conditions: 10 min at 94°C, followed by 37 cycles of 30 s at 94°C, 30 s at 60°C and 70 s at 72°C, then 10 min at 72°C. Samples were then kept at 12°C. The PCR products were detected by electrophoresis on agarose gel containing ethidium bromide.

### The expression of *SeHex *and *SeSP1 *in starvation treatment by RT-PCR amplification

The day-2 fifth instar larvae were used as experimental insects in the treatment of starvation. In the experiment of starvation, all of the insects were divided into two processed groups and a control group. Two experimental group insects were starvation with 6 h and 12 h, respectively. Followed by these insect continue to rear at 25 ± 1°C with an L14:D10 photoperiod using an artificial diet. The insects were observed at 0 h (before treatment), 6 h, 12 h, 24 h, 36 h, 48 h and 72 h after treatment. Every three to five lively larvae were randomly selected and stored at -80°C for subsequent RNA extraction. The way of PCR reaction just as the previous.

### Injection of dsRNA into *S. exigua *larvae

dsRNA for *SeHex *and *SeSP1 *genes was prepared according to methods established in the State Key Laboratory of Biocontrol of Sun Yat-sen University [60]. The T7 RiboMAX™ Express RNAi system (Promega, USA) was used for synthesis. Day-2 fifth instar larvae were used for injection experiments. For each treatment, 5 μg of dsRNA dissolved in 5 μl of DEPC water was injected into the side of the thorax of a larva using a 10- μl syringe (Hamilton) and the injection point was sealed immediately with wax. Control larvae were injected with an equivalent volume of dsRNA corresponding to a *GFP *gene or were not injected. Each group comprised 30-40 individual larvae and semi-quantitative RT-PCR and survival analyses were carried out in triplicate.

### Data analysis and insect survival

Insect survival and morphological changes were recorded every 12 h. The insects were observed at 24 h (day-2 5th instar larvae), 36 h (day-3 5th instar larvae), 48 h (pre-pupae), 60 h (pupae) and 204 h (adult) after injection. To assess the effect of a treatment, ANOVA was performed using the cumulative percentage of abnormal and dead larvae as the dependent variable and treatment (dsSeHex, dsSeSP1, dsGFP or no injection) as the independent variable. Post hoc Duncan's tests were used to determine differences among groups when treatment effects were detected. Results were arcsine square-root transformed before analysis to correct for the non-normal distribution of percentage values.

### Semi-quantitative RT-PCR analysis of gene silencing

Insects were observed at 12, 24, 36, 48, 72, 96, 120 and 168 h (pupae) after injection. Larvae in three states (before death, still living and less vital) were randomly selected at each time point and stored at -80°C for subsequent RNA extraction. Total RNA was extracted from individual larvae using the acid guanidinium thiocyanate-phenol-chloroform method [61,62] and converted to cDNA using AMV reverse transcriptase (Takara). The Hex-FP/Hex-RP or SP1-FP/SP1-RP primers were used to amplify all three cDNAs in the same PCR reactions. Pilot experiments demonstrated that 22-24 cycles were optimal for linear amplification of the PCR products, and this protocol was then used in subsequent experiments. PCR amplification was performed in a 25- μl reaction mixture under the following conditions: 10 min at 94°C; 22-24 cycles of 1 min at 94°C, 1 min at 60°C and 1 min at 72°C; and a final 10 min at 72°C. The PCR products were separated on a 2% agarose gel and transferred to a Hybond-N^+ ^nylon membrane. Hybridization, washing and signal detection of the blots were similar to methods previously described [59].

## Authors' contributions

BT carried out all experiments and wrote the manuscript. SGW was involved in helping to draft the manuscript and revising it critically for important intellectual content. FZ conceived the project and supervised the experiments. All authors read and approved the final manuscript.

## Supplementary Material

Additional file 1 **Figure S1 Alignment of the amino acid sequences deduced for (A) *Hex *and (B) *SP1 *genes in insects**. The amino acid sequences deduced for insect *Hex *and *SP1 *genes were aligned using Vector NTI 9.0 software. Highly conserved regions are yellow and sky-blue. Click here for file
