# Supplementary material for: Two storage hexamerins from the beet armyworm Spodoptera exigua: Cloning, characterization and the effect of gene silencing on survival
Source: BMC Mol Biol. 2010 Aug 31;11:65. doi: 10.1186/1471-2199-11-65 (PMC2939506; doi:10.1186/1471-2199-11-65)
Supplement: Additional file 1 — Figure S1 Alignment of the amino acid sequences deduced for (A) Hex and (B) SP1 genes in insects. The amino acid sequences deduced for insect Hex and SP1 genes were aligned using Vector NTI 9.0 software. Highly conserved regions are yellow and sky-blue. [file 1471-2199-11-65-S1.PDF]

Figure S1

A

```
GmHex   (1)   MGRLVLCVLALLVGGGISDPVKKLQR--TVDQTVLDRQYKLLTLFFHPHEPIHIKEQQEIAASWDLEKNIGLYENATAVH
HaHex   (1)   MGRLGLCVLALLVAGGHSDPVRSNLSQKAADPVFLQRQLDLMVLYFHLLEPNHLDSCQAIAKSWSLERNIEHYSNVTAVT
HceHex  (1)   MALLSLFVLALIFAPGLCDPVRTVDS-----VEFAKKQIDISLLYYHAREPNHVSKCITISASWSIENNINHYKNETAVK
OfHex   (1)   --------------------------------------------------------------------------------
SeHex   (1)   MARLALCVLALLVAGALSSPDRSNVSPKVADPVFLQRQVDLMILFFHLHEPNHPESWKQISKSWSLEKNIEHFSNVTAVT
TnHex   (1)   MARLVLCALALLVAGGLGVPVRNNVPQKPADPVFAKRQMDLMTLFFHVLEPNYIEANKVIVNTWSLEKNIEHFSNVTAVT

GmHex   (79)  LTIQMLHNNYQVPRGVPFTVLESVHRFEISVYYSLLYSAKTYDTFYKTAVFLRQHVNENLFVNVLSVVILHRSDTQDIRI
HaHex   (81)  TYIEMLEHQWILPRAVPFSLLQAEHRFGAVTLYNVLISAKDYDTFYKTAVYVRDLVNENLFAYVLSIAILNRPDTQGIYI
HceHex  (76)  IWIEMKKQNWLLPLSVPFSPLNPSHQFEAITLFNILYSAKDYDTFYKTTVYMKDRVNQDLFIYVLSILHIHRSDLEGYAI
OfHex   (1)   ----MIEHNWLLPQGLPFSVLEHEHIYQVQTYFNLLYSAKTFDTFYKTAVYLRDNINEYIFVYVTTVAIYHRADTQGIIV
SeHex   (81)  TYIEMLEHKWVLPRAVPFSLLESGHKFEAVTLYNVLYSAKDYDSFYKTAVYLRERVNEHLFAYVLGVAIVNRPDTQGIYI
TnHex   (81)  TYIKMLENQCCVPRAVPFSILEDEHKFEVVTLFNVLHSAKDYDTFYKTAVYVRDRVNPDLFSYVLSAVIVNRPDTKGIYI

GmHex   (159) PPIYDVFPSYFHNGEIYDDSPKNNYSWSTNVRTLPINICLGEQCCIRHNETAWPYYCNTESMPVSYFTHDVTLNALYYNI
HaHex   (161) PRLPEVFPSYFYNGEIMTTAQRINTHGQNMVEHYPSTYKWDNNVVIRWNATIWPYLRSET-MPLAYFTHDFSLNTFYYNL
HceHex  (156) PPIYEVLPEYFNNGEIMTTAQRIGVHGSHMIEYYPSTYKWDNSVVIRWNTTVWPYQCQS--TPMSYYLHDYSLNAHYYYH
OfHex   (77)  PPLYEIFPSFFNNAEIMTTAQRITTHGKHWVEHYPSTYVWDNNVVVRWNSSVWPYYSKE--MPVSYFTHDYVLNNYYYNI
SeHex   (161) PRLHEVFPSYYNNGEILTTAQRINTHGHRLVESYPSTYKWDHNVVIRWNATIWPYSNDHS-TPVAYFTNDFGLNTYYYNY
TnHex   (161) PRVFEIFPSYFNNGEIMTTAARINTHGDRLVDFYPSTYKWDKNVVIRWNPHLALLQQPE--HTYSLLHSRLQPQLILLQR

GmHex   (239) KLAYPIWLRSDACAIKE-KRGELFFFWNKQLLARYYMERLSVGLGEIPELGLNEVEEGYVSGLLYHNGIPYPVRPNHLVL
HaHex   (240) HLAQPSWLHSEVLPVNKHRRGEWFWFLHKQILTRYYMERLSNGLGEIPELGHEIVQYGYASGLLYHNGVPFPARPNYFNL
HceHex  (234) HLTYSKWLGGDVIPLLKERRGEWYWFVHKQLVTRYYMERLSNGFGEIDELSGDVVNEGYNFGYMYHNGIPYPVRPNHFHL
OfHex   (155) HALYPPWLGNEVVPLIKDHRGEWFWFIHKQILARYYMERLSNGLGEIPELGLDIVKQGYSSGLAYHNGIPYPVRPNYLYL
SeHex   (240) HLAQPSWLHGEVLPLHKGRRGEWFWFVHKQIVARYYMERLSNGLGEVPELGHNIVKEGYNPGLLCHNGIAFPVRPDYYHL
TnHex   (239) SLSLPGWLQTEALPVNQHRRGEWFWFLHKQLIARYYMERLSNGLGEIPELGHETVKDGTTRSFGTTMEFSSPVRPNNFNL

GmHex   (318) NHQTWHAEAIEEIEVYENRIRDMIDQGFYITNTGEHVSINSPDSIDVLGRLIEANVDSPNVQYYKDFISIWKKVLGNSLV
HaHex   (320) DQP-QLVNEIQEILDYERRIRDAIDQGYVVNHLGEHIDICAPEAIEILGSIIEANVDSPNAKYYKDFISIWKKVLGDSIV
HceHex  (314) DHP-EHIGEIEKIKNYERRLRDAIESGYIINSAGDHVDISSPEAIDILGRLIEAGVDSPNVHYYKDFISSWKYVLGNSLW
OfHex   (235) QQP-WLVHKIDQIVDYERRMYDVIDSGLYVTPAGKYVNVHHPEAIDVIGRLIEANVDSPNKKYYKDFISVWKNLLGNSNV
SeHex   (320) DRP-VLVHALQKIDDYERRVRDAIEQGYVLNHLGERIDISSPEAIEILGSVIEANVDSPNLKYYNDFISLWKKVLGNSIV
TnHex   (319) DQP-EFVNEVEQIYDYERRVRDAIDQGYVLNHLGERIDISAPEAIEILGRVIEANVDSPNVQYYKDFISVWKKVLGNSLV

GmHex   (398) HESVAFN---GIPLVVPSVLEQYQTALRDPAYYMIMKRVLKLFNLWHEHLPHYTTKELSVPSVKIEKVEVDKLLTYFEYT
HaHex   (399) QDNQYHN--NYVPLVVPSVLEHYQTALRDPAFYMIWKRVLGLFQMWQEKLPLYKKEELALPQVAIQKVDVDKLVTYFEYT
HceHex  (393) YRHNYNNPGSATPVLIPSALEHYQTALSDPAFYMIWKRVLKLFSLWHHRLPQYKNEELALTDVTIEKVEVDKMVTYHEYT
OfHex   (314) HEPIYWKG---IPLVLPSALEHYQTALRDPAFYMIWKRVLHMFTLWQEKLPYYTKEDLYFPGVKVDNVVVDKLVTYFDDY
SeHex   (399) HEHQYHH--HVVPLVVPSVPEHYQTALRDPAFYMIWKRVLGLFQLWQEKLAPYKREQLAVPQVAIEKVDVDKLVSFFEYN
TnHex   (398) HEHQYFH--HYIPLVVPSVLEHYQTALRDPAFYMIWKRVLGLFQQWQEKLPHYKPEELAMPQVAIEKVDVDKLVTYFEYS

GmHex   (475) NFNVTNHLHLNEIECNNVINTKSVLVQRTRLNHKVFTVR-VNVKSGVAKHVTVRFFLAPKYDSVGNEIPLNVNTQNFLLI
HaHex   (477) YLNVSSHLHMNQDEVKGYYDQVSVLVQQPVLNHKRFQVR-VNVKSEVAKTVLVKFFLAPKYDSQGYEIPLHINTQNFMQL
HceHex  (473) YTNISAAIHMNEVQSQLMYDKESVLVQYARLNHKKFNIR-VQVSSKVTKKVEVKFFLAPKYDSRGFEIPLHANSENFFLL
OfHex   (391) LMDITNAVILTEDELKKTTSDMKLFVRKRRLNHQPFKVTRSMFSLTKAADCVVRFFLAPKYDNVGRLIDINMNRLNFVEL
SeHex   (477) YLNISSFLHMNEEEAKELYDQVSVLVQHPRLNHKKYQVR-VHVKSEVAKTVLVKFFLAPKYDSHGYEIPLHVNSHNFLQI
TnHex   (476) YMNVTSGLPMNVEEAKELYDQVSVLVQHPRLNHKKFQVR-VNVKTEVAKTVLVKFFLAPKYDSHGHEIPLHVNSYNFMQL

              561                                                                          640
GmHex   (554) DIFNYELKEGDNLITRVSSDNLLVTDEIDSASVLFNKVDSALQGHGQYMLNMKQNILKTPRHLLLPKGRVGGMPFVLMVY
HaHex   (556) DEFTYDLPAGECTITRDSIDTSG--KKWVSGIEIYEAVEKAVQGKGQYTIDP--NMEKLAEHLMLPKGRVGGMPFVLMVY
HceHex  (552) NHFVHELTAGENVIVRESTQNSFTVDDLESAYEIYMKAQNIVEGKKETLKERTKHLDFFPHHLLIPKGRVGGMPFVLMVY
OfHex   (471) DSFLYKLTTGQNVIIRNSYDMHNLVRDRVMTRDLWKKVDTVTDFRDLLVKDLRNYHTGFPTRPLLPKGIVGGMKMMLYVI
SeHex   (556) DEFVHDLPAGESVIARDSVDTS---KVWDTANNVYYAFEKSLQGDKQFNMEQLENMESLVQHLTLPKGRVGGMPFVLMVY
TnHex   (555) DEFVYDLPQGESVITRDSVETTG--NEWTTSYQVWDQAEKASR-EKHLSTSTRRQHAQVGSHLLLLKVVSVHALRPDGLH
```

B

```
SeSP1     (1) ---MRVLLVLAAVALAAANVVKDDTNVFIGKDNMVNFDIKMKELCIMKLLNHTLQPTMYDDVREVAREWTIKDNVDKYLK
AaSP1     (1) MRSFSVLVVAAALAVASAAFVPNTS-----QVKYADKEWLHKQEDLLLLFRHLYQKDWNPQLVAYAKNFLVEKSFHLYDN
RsSP1     (1) -MNTALLFATVVAVLVCGAFSDHHV-----GKKVADKPFLMKQKNILGLLHRIHQDNVFKEQVDVGNTYDIEAHISNYKN
TcSP1     (1) ---MRFIVVAILGCCALALAFP--------ERKITNKQFLERQRDILRLFRHINQPSYYKDHQEIAQSFHLHDHYDHYTK

SeSP1    (78) AEVVRDFIDAFKMGMLPRGEIFVHTNELHMIQAVKVFKILYSAKDFDVFMRTACWLRERINGGMFVYALTACVFHRADCR
AaSP1    (76) EDAAREFWTYYNHGMLPKGEVFSVFNEVHREQAVALFHLFYYAKDWDTFYKTAAWARYYMNEGMFVYALTVAVTHRQDFA
RsSP1    (75) TKVVKEFISYYKKGMLQRWEPFSVYYKTHLEQAISLFELFYFANDFDTFYKTACWARDRVNPLMFWYSFTAAVLHRDDTT
TcSP1    (70) PEIAKHYHQIYEYGLLPRGEVFSVFYEEHLQQAIALYKLFYYAKDYDTFYRTAVWARQHVNEGVFLYSFSVAIVHREDTY

SeSP1   (158) GITLPAPYEIYPYFFVDSHVINKAFMMKMTKAANDPVLMNYYGIKVTDKNLVVIDWRKGVRRTLSQNDRISYFTEDIDLN
AaSP1   (156) GFILPAPYEIYPYYFINTEVIQKAQQYKMQGYYGMKKVGEVYTAVVTSN-------YTGWYMHTNPEQKISYFTEDIGLN
RsSP1   (155) DVMMPPPYEVYPYFFVDSDIIQKAYKYWMMHVG----TPEHHTYIIPMN-------HTMKSKEN----LLYYFTEDVGLN
TcSP1   (150) GIVLPPIYEIYPHYFYNNEVIQEAYRYKQQYYN------QEHGYTINAN-------YSGFYLNLHPEQSLSYFTEDVGVN

SeSP1   (238) TYLYYSHMSYPYWMNHDMYAVNKERRGEIMSYATMQLLARLRLERLSHEMCDIKPIMWNEPLKTGYWPKIRLHTGDEMPV
AaSP1   (229) TYYYYFHIDYPFWMGGKEFGLYKDRRGELYLYEHQQILARYYLERLSNDLGHIPKFSWYWPVKTGYYPDLQYYNGHTFPN
RsSP1   (220) AFNMYYRMYYPSWFNVTEYGHKFDRRGEMFLYVQHQLYARYSLERMSNGMPEVQPFVYNKPLKTAYNPNLMYHTGQEMPP
TcSP1   (217) SFYYYYNIYYPHWLGGEDFDFAHDRRGEQYYYVYQQILARYYLERLSNDFGEIPFFNYEVPFENGYYPLLQYPNGLFFPQ

SeSP1   (318) RS-------NNMILVTKGN----LKWKRMLDDVERKLRDEILNGKIERRDGTVISLKKP-EDIEYLARMVLGGMGLVSDD
AaSP1   (309) R-------DNFYFVNQESN----YYDIQLVDDYEHRIREVIDRGFLVKSDGKMINFTTP-EAIDYLGNLIQSNP-DSYDT
RsSP1   (300) RP--------SDKLVTNFD----TYTIADIKNYERRVADAIDFGYVKDEHLKIHSMYEDNNGIDYLGQMIEGSY-NSPHY
TcSP1   (297) RPNYAKLYEYFYNYGQKYGNNRYAYSYTFVQDYERRIRDAIDRGYVFSHDGQRINLFSE-DGVNILGNLIESNP-DSPDR

SeSP1   (386) AMFMHMMHLMKRLLSYNVCDFDKYTYVPTALDMYTTCLRDPVFWRLMKRVTDVFVLFKKMLPKYTRDDFDFPGVKIDRFT
AaSP1   (376) RYFKYLSMFSRIIMGAAVEPVEAYQVIPSVLEHFESALRDPVFYQIYKRIIQYYYQFKNHLPAYTYEELYFPGVKIDDVV
RsSP1   (367) YYYGSLFHFYRMMLGHMMDPFHKHGLAPSALEQPETALRDPAYYQLYKRMYHLVNKYKDRLPRYTHEQLWFEGVTVENVD
TcSP1   (375) HFYGALHVYARHLLGYSSQPLDKYHVAPSALQHYETSLRDPAFYQFYKRIVLYFQKYKSYLPSYTEHDLHFQGVEVKSVE

SeSP1   (466) TDKLVTFMDEYDLDITNALYLDDAEMK-----------KKRPDMLMVARMRRLNHHPFKVTVDVTTDKTVDAVVRIFIGP
AaSP1   (456) IDKLVTYFDRFDVDITNAIDIEPEPYVEGKYDGFGEIEYKPDPVVIKARTIRLNHKPFTYKISVTSEKPVKTVVRVFIGP
RsSP1   (447) VGKMYTYMENFEFSLGGTIYVAK--------------EEDMLGVNLHVRQPRLNHKPFTYKIEVNSEKAVDAYVRVFLGP
TcSP1   (455) FDRLVTYFDHFYTDISNAVYVTP------Q-------EYDSEKVQVRVRQYRLNHKPFTYRVHVSSDKEQQAVVRIYLGP

SeSP1   (535) KYDCLGRLMSVNDKRLDMVEIDTFKYKLETGKNTIVRNSVEMHGVIERRPWTRRILNNMRDTVGMISKTVDVESWWYKTR
AaSP1   (536) KYDEFGAQYKLNENRENYYELDYFVFDLVPGKNVITRNSLSFNGYVKDRTSFYELYKKVMLGYKGDEK---FPLDMSEAH
RsSP1   (513) KHNYLDEEWDLNERRHFFVEMDRFRHHVPAGKSVIERNSHDSSIISPTPDSYRTFVKKVQDAYDGKTQ---YFIDKSHNY
TcSP1   (522) KYDEYGRYINISHNRLNFVEVDHFKYQLKVGENVIERNSHQNYFYQNDRTSYRQLYKQVLGALNGNGE---FNVNANEAY

SeSP1   (615) VGYPHRLLLPLGRLGGFPLQMYVIITP--VRTNLLLPTVDMNLMKERRTCRWSVCFDTMPLGFPFDR--KIDMTKFFTQN
AaSP1   (613) CGFPNRLMLPKGKKGGMPFQFFFMLSPHYAPEVPQYTGFDP--VVSCGVGSGARYFDKLPFGYPFDR--KIDETYFHVPN
RsSP1   (590) CGFPENLLLPKGQKGGETFTFYVIITPYVKQDEHDFEPYDYKAFSYCGVGQDRKYPDDMPLGFPFDR--QIHSKDFYTTN
TcSP1   (599) FGFPRRFLLPKGNYGGQEFQFFVIVSP-YVPYKYHQEGYDASKYYYPRVGSGAHHIDNYAFGYPFDRPIHYDQIFHNVPN
```
